# Biological Evaluation of Noscapine analogues as Potent and Microtubule-Targeted Anticancer Agents

**DOI:** 10.1038/s41598-019-55839-8

**Published:** 2019-12-20

**Authors:** Vartika Tomar, Neeraj Kumar, Ravi Tomar, Damini Sood, Neerupma Dhiman, Sujata K. Dass, Satya Prakash, Jitender Madan, Ramesh Chandra

**Affiliations:** 1 Department of Chemistry, University of Delhi, Delhi, 110007 India; 2 BioMedical Engineering Department, Faculty of Medicine, McGill University, Montreal, Canada; 3 Amity Institute of Pharmacy, Noida, U.P India; 4 BL Kapur Hospital, New Delhi, 110005 India; 5 Chandigarh College of Pharmacy, Mohali, Panjab India; 6 Dr. B. R. Ambedkar Center for Biomedical Research, University of Delhi, Delhi, 110007 India

**Keywords:** Biochemistry, Chemical biology

## Abstract

In present investigation, an attempt was undertaken to modify the C-9 position of noscapine (Nos), an opium alkaloid to yield 9 -hydroxy methyl and 9 -carbaldehyde oxime analogues for augmenting anticancer potential. The synthesis of 9-hydroxy methyl analogue of Nos was carried out by Blanc reaction and 9-carbaldehyde oxime was engineered by oxime formation method and characterized using FT-IR, ^1^H NMR, ^13^C NMR, mass spectroscopy, and so on techniques. *In silico* docking techniques informed that 9-hydroxy methyl and 9-carbaldehyde oxime analogues of Nos had higher binding energy score as compared to Nos. The IC50 of Nos was estimated to be 46.8 µM signficantly (P < 0.05) higher than 8.2 µM of 9-carbaldehyde oxime and 4.6 µM of 9-hydroxy methyl analogue of Nos in U87, human glioblastoma cells. Moreover, there was significant (P < 0.05) difference between the IC50 of 9-carbaldehyde oxime and 9-hydroxy methyl analogue of Nos. Consistent to *in vitro* cytotoxicity data, 9-hydroxy methyl analogue of Nos induced significantly (P < 0.05) higher degree of apoptosis of 84.6% in U87 cells as compared to 78.5% and 64.3% demonstrated by 9-carbaldehyde oxime and Nos, respectively. Thus the higher therapeutic efficacy of 9-hydroxy methyl analogue of Nos may be credited to higher solubility and inhibitory constant (K).

## Introduction

Most of the synthetic antineoplastic therapeutic modalities available today are immunosuppressive agents and exert several side-effects^1,2^. Plants are playing a key role in generating the newer antineoplastic agents^3^. Approximately, 70% of currently available antineoplatic agents are derived from either plants or other natural sources such as marine organisms and microorganisms^4^. Vincristine, vinblastine, and podophyllotoxin are clinically available plant derived anticancer agents^5,6^. Each anticancer agent has its own mechanism to induce apoptosis in cancer cells. With several successful anticancer drugs such as paclitaxel^7^ and docetaxel^8^ in the market and numerous compounds in clinical trials, armamentrum of antimitotic agents represent an important class of antineoplastic agents.

**Figure 1:**
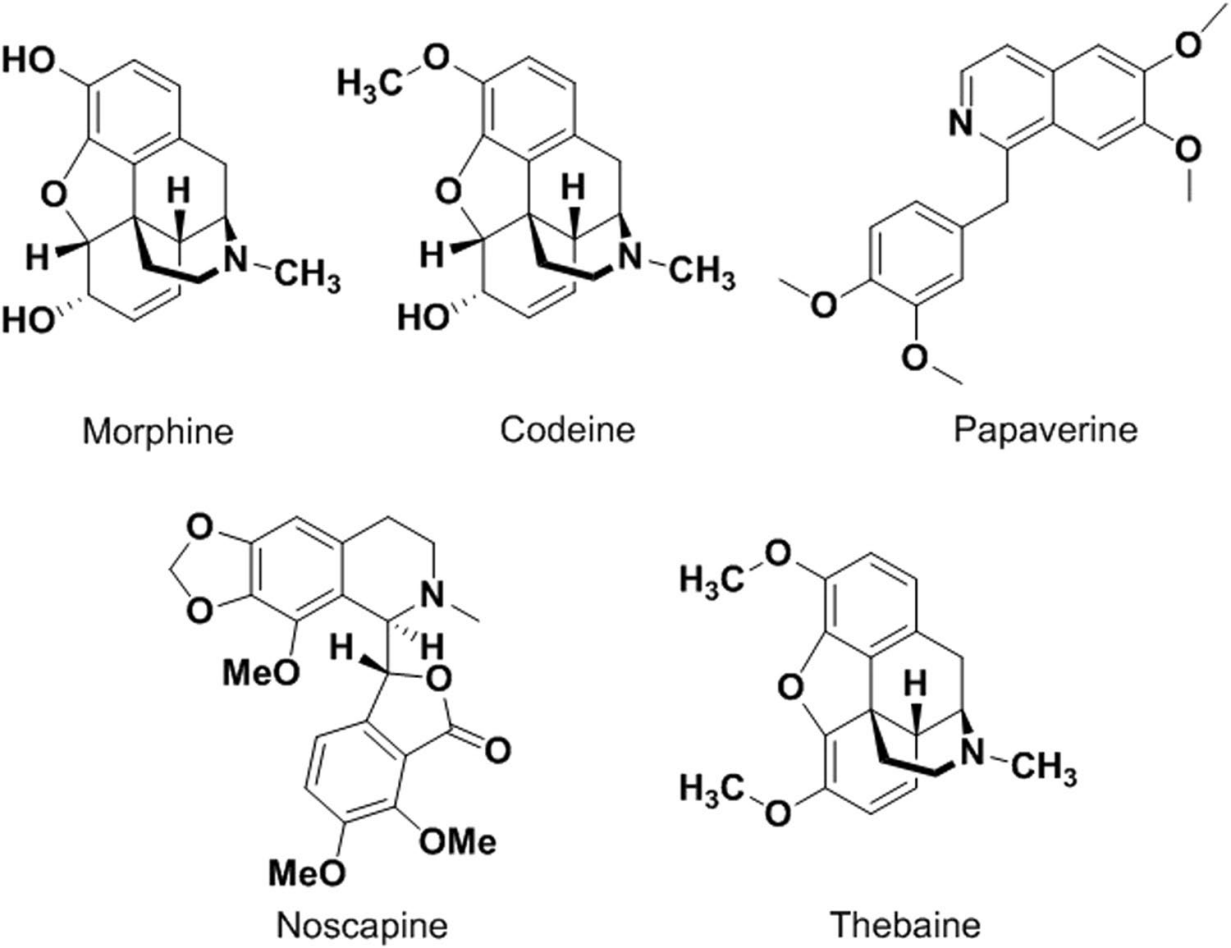
Schematic representation of active ingredients of opium.

Noscapine is one of the ingredients in Papaver somniferum (opium). It was firstly isolated from Papaver somniferum (opium) in 1817. It is one of the most abundant opioids present in the opium plant (up to 10% of the total composition) after morphine. It also known as Narcotine, Nectodon, Nospen, Anarcotine and (archaic) Opiane and occurred in is the (-)α isomer which has S, R stereochemistry (S stereochemistry at phthalide-carbon and R at isoquinoline-carbon). Noscapine is structurally and chemically different from other opium alkaloids such as morphine (Fig. 1**)**, codeine, thebaine, papaverine and narceine. It was used as anti-tussive agent but later on Ye and co-workers have discovered its anti-neoplastic properties in 1998. It works as a tubulin inhibitor by binding stoichiometrically with tubulin, causing a change in its conformation.

**Figure 2:**
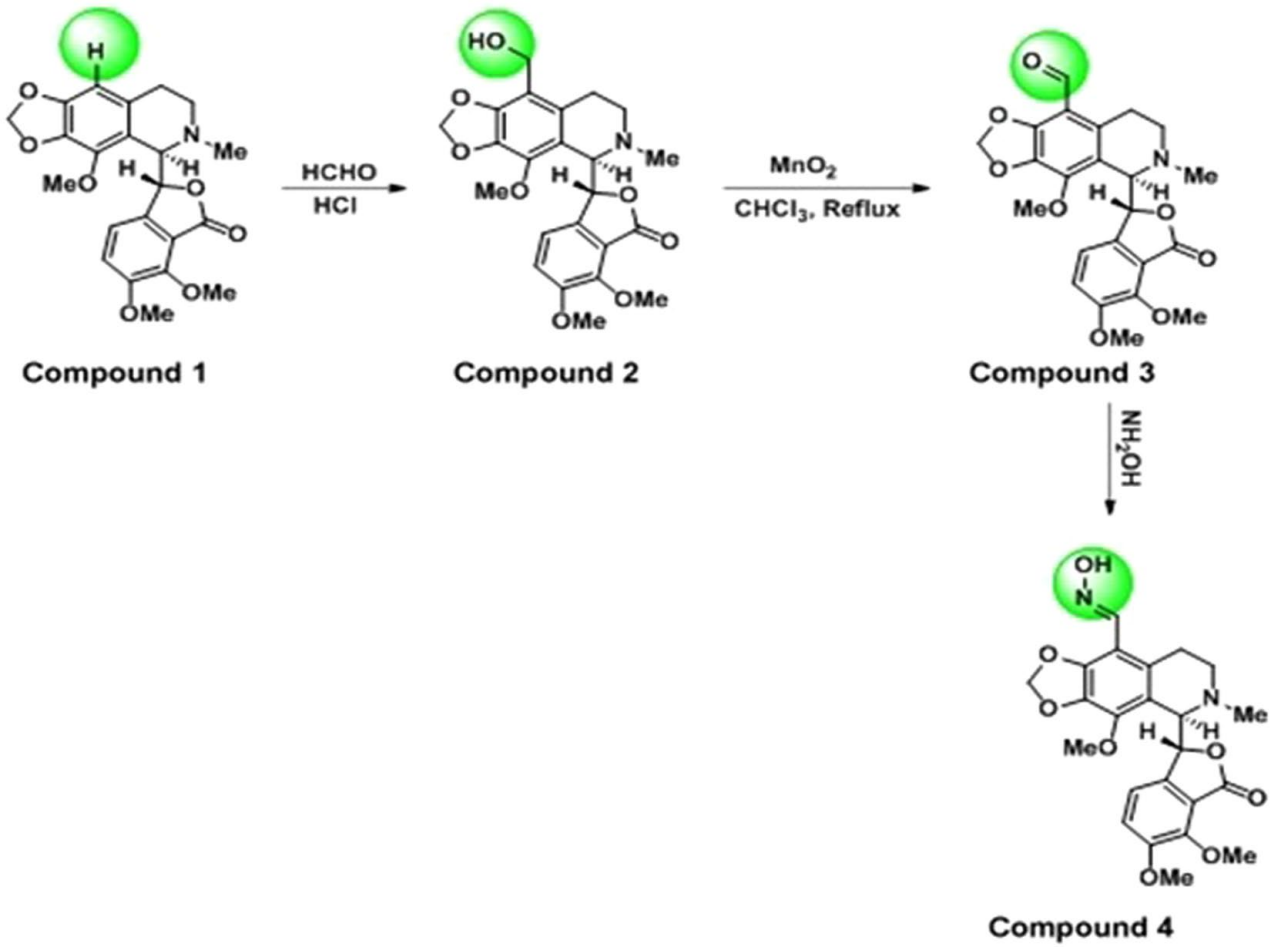
Schematic representation of synthesis of 9-hydroxy methyl (Compound 2) and 9-carbaldehyde oxime (Compound 4) analogues of noscapine (Nos) using Blanc reaction and oxime formation method, respectively.

Our laboratory is continuously working on development of plant derived anticancer agents such as noscapine from the last 20 years. We have reported the anticancer activity and synthesis of several potent analogues of noscapine, a plant derived anticancer agent^9–12^. Structure-activity analysis demonstrated that modification of the proton at C-9 position of the isoquinoline ring in noscapine (Nos) can be done without affecting the tubulin binding activity. In this context, brominated (9-Br-Nos) and reduced brominated analogue of noscapine (Red-Br-Nos) exerted 5–40 folds more cytotoxicity as compared to parent compound, Nos in cancer cells^11,12^. Identical to *in vitro* data, Nos, and halogenated analogues of Nos also displayed potent antitumor potential in preclinical xenograft model^13–15^. Apart from halogenated analogues, 9 (4-Vinylphenyl)^16^, 9′-amino^17^, 9′-nitro^18^, 9′-azido^19^ and cyclic ether halogenated analogues^20^ have also demonstrated good anticancer potential as compared to Nos. On the other hand, O-alkylated and O-acylated analogues of Nos represented the second-generation noscapinoids that were customized by transforming the benzofuranone ring^21^. In addition, third-generation noscapinoids were synthesized by modifying the nitrogen atom of the isoquinoline ring^22^. Therefore, in present investigation, two novel analogues namely, 9-hydroxy methyl and 9-Carbaldehyde oxime (Fig. 2) were synthesized by using C-9 position of Nos. The synthesis of both the analogues was confirmed by fourier-transforms infrared (FT-IR) spectroscopy, nuclear magnetic resonance (^1^H NMR) spectroscopy, carbon nuclear magnetic resonance (^13^C NMR) spectroscopy and mass spectroscopy. *In silico docking* was employed using Lipinski rule of five^23^ to determine the tubulin binding efficiency of 9-hydroxy methyl and 9-carbaldehyde oxime analogues of Nos. The *in vitro* cytotoxicity^24^ and apoptosis assays were utilized to examine the therapeutic potential of synthesized compounds against human glioblastoma cell line, U87 and U251 resistant glioblastoma cell line.

## Results and Discussion

### Synthesis and confirmation of 9-hydroxy methyl and 9-carbaldehyde oxime analogues of noscapine

Our laboratory is actively engaged in development of novel analogues of opium alkaloid, noscapine (Nos)^9–12^ that have better anticancer potential. Nos (Fig. 2, **Compound 1**) carries two ring systems namely, isoquinoline and benzofuranone ring that are linked by a labile C-C chiral bond. Moreover, these ring systems hold numerous susceptible -OCH3 groups. In this way, it is challenging to achieve selective substitution at C-9 position of Nos without disrupting the labile groups and C-C bonds^25^. In present investigation, we have synthesized the hydroxy methyl derivative (Fig. 2, Compound 2) of Nos using paraformaldehyde (40% formalin solution) and dry HCl gas via Blanc reaction^26^. The excess amount of concentrated HCl or longer reaction time was avoided in order to protect the product from hydrolysis. Subsequently, compound 2 was oxidized to compound **3 (**Fig. 2**) via** oxidation reaction^27^ in presence of MnO2. Here, MnO2 was used as oxidizing agent. Other oxidizing agents like chromic acid, KMnO4 and acetic acid were also employed for the oxidation of compound 2; however, each oxidizing agent has its own limitations. Compound **3** was converted into compound 4 (9-carbaldehyde oxime) using hydroxylamine HCl at 0 °C^28^ (Fig. 2, Compound 4). In this fashion, the substitution was selectively took place at C-9 position of the Nos. Substitution was further confirmed by disappearance of the aromatic singlet proton of C-9 at 6.30 ppm in the ^1^H NMR spectrum of the product. ^13^C NMR and mass spectroscopic data also supported the modifications at C-9 position of Nos.

### 9-hydroxy methyl and 9-carbaldehyde oxime analogues of noscapine displayed potent tubulin bidning efficiency

**Figure 3:**
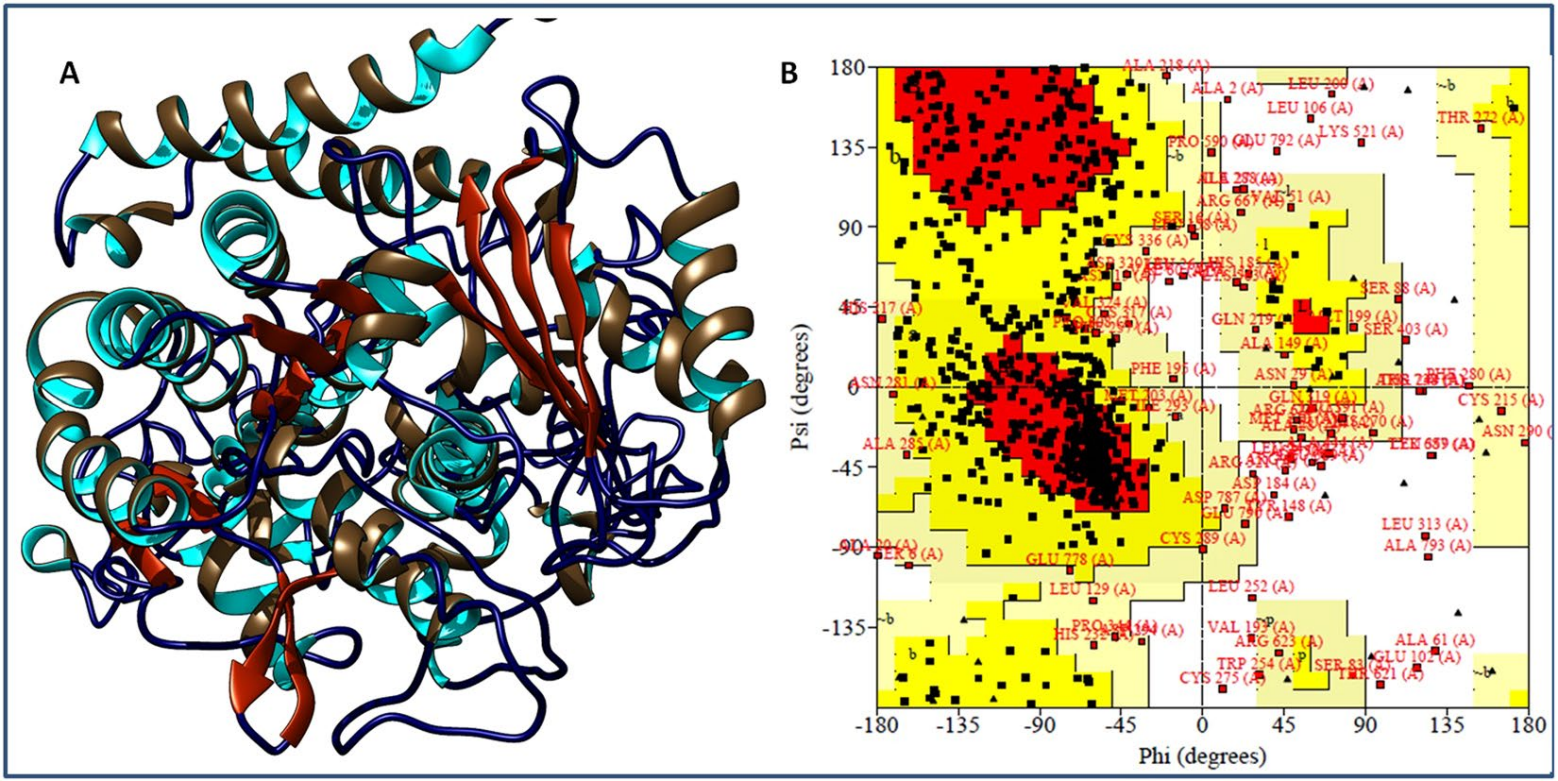
(**A**) 3D Structure orientation of binding groove of tubulin protein. (**B**) Ramachandran plot of the tubulin protein lying under the allowed region of Ramachandran plot.

**Figure 4:**
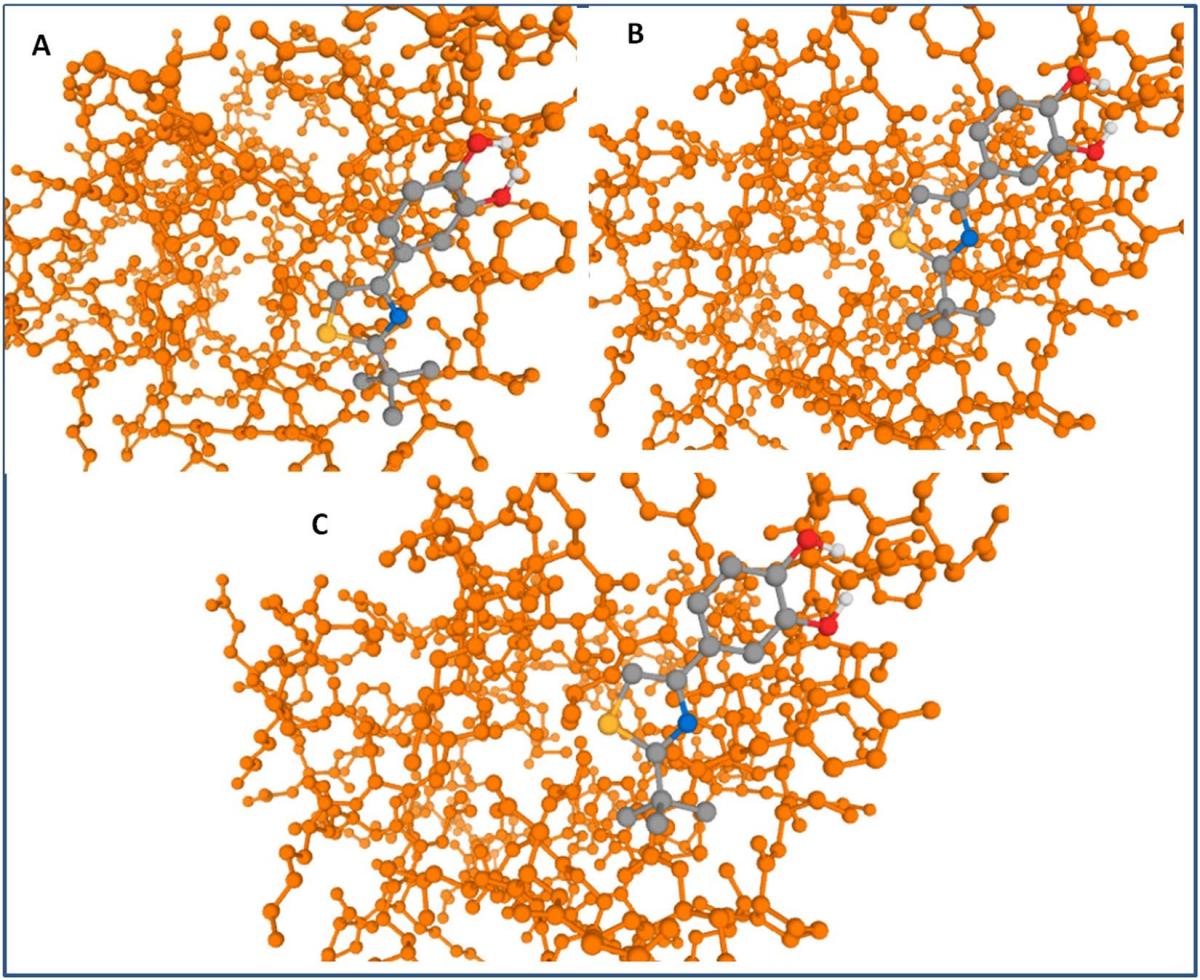
**(A)** Depiction of interaction of noscapine (Grey in color) in binding groove of tubulin protein (Orange in color), (**B**) Binding of 9-hydroxy methyl noscapine (Grey in color) with tubulin protein (Orange in color) and (**C)** Binding of 9-carbaldehyde oxime noscapine (Grey in color) with tubulin receptor protein (Orange in color).

*In silico* docking analysis was carried out to predict the physicochemical and tubulin binding efficiency of synthesized analogues. For molecular docking, three-dimensional structure of tubulin protein (PDB ID: 1SA0) was retrieved from protein data bank and it consists of only α- and β- chains. Basically, it is a tubulin-colchicine stathmin like domain complex protein at 3.58 A° resolution^29^. Furthermore, two subunits α- and β- chains of tubulin are made up of two chains A and C chain as well as B and D chain, held through two regions located on opposite ends (Fig. 3A,B). The α-chain contains the sequence of 451 amino acids, and β-chain is composed of the sequence of 445 amino acids. Tubulin protein receptors bind through extracellular domain region to any drug or regulator. Extra cellular domain region in addition to cytoplasmic region of around 100 amino acids each with short 13 amino acid residue actively participate in stabilizing the protein structure. Tubulin protein receptor of human origin was extracted from the tubulin-colchicine protein complex and analyzed for its conformations using Ramachandran plot. Tubulin protein stereochemical properties were analyzed using Rampage server^30^ that displayed 91.1% of residues of the protein structure. Furthermore, 78.8% residues were found in the favored region and 12.3% residues were calculated in the allowed region. However, a number of small residues, about 8.9% were measured in outlier region (Fig. 3A,B). Ramachandran plot clearly indicated the stable conformation of tubulin protein receptor that can be further used for molecular modeling studies. Molecular docking studies were performed to determine the binding interaction of Nos, 9-hydroxy methyl and 9-carbaldehyde oxime analogues of Nos (Fig. 3A,B and Table 1). The tubulin protein was docked with Nos and 9-hydroxy methyl as well as 9-carbaldehyde oxime analogues of Nos using the autodock tool (Fig. 4A–C). Molecular docking analysis demonstrated that 9-hydroxy methyl and 9-carbaldehyde oxime analogues of Nos had higher binding energy score as compared to Nos (Table 1). Nos displayed the binding energy score of −10.20 kJ/mol with inhibitory constant of 17.8 µM. On the other hand, 9-hydroxy methyl and 9-carbaldehyde oxime analogues of Nos resulted in docking score of −10.73 and −10.83 kJ/mol, respectively (Table 1). This justified that 9-hydroxy methyl and 9-carbaldehyde oxime analogues of Nos have higher binding efficiency and inhibitory constant on the fastening locations of tubulin in comparison to parent molecule, Nos. Molsoft software was exercised to assess the drug likeliness properties of Nos and synthesized analogues. In addition, Lipinski rule of five was adopted to assess the pharmacological efficacy of the synthesized compounds. Lipinski properties govern the pharmacokinetic attributes of therapeutic moieties. ADME properties are currently being widely used for a chemical compound to be a novel lead. Nos had drug likeliness score of 0.55 with molecular formula of C22H23NO7. In addition, molecular weight (Mw) of 413.15 Da, 8 numbers of hydrogen bond acceptor (HBA), partition coefficient (log *P*) value of 2.36, solubility coefficient value of −2.9 and two stereo centers of Nos were also estimated. Consistently, 9-hydroxy methyl and 9-carbaldehyde oxime analogues of Nos had better drug likeliness scores of 0.86 and 0.79, as compared to Nos. 9-Hydroxy methyl analogue of Nos displayed the molecular formula of C23H25NO8 with Mw of 443.16 Da. Moreover, HBA numbers were calculated to be 9 in addition to 1 number of hydrogen donor group (HBD), Log P value of 1.54 and Log solubility value of − 2.74 moles/L (Table 1). Correspondingly, analysis of 9-carbaldehyde oxime analogue of Nos showed the molecular formula of C23H24N2O8, Mw of 456.15 Da, HBA value equivalent to 10, HBD value of 1, LogP value of 2.16 and solubility of −3.30 m Mg/L (Table 1). The pharmacokinetics properties of the noscapine derivatives (9-hydroxy methyl noscapine and 9-carbaldehyde oxime noscapine) were also assessed. It was seen that both 9-hydroxy methyl noscapine and 9-carbaldehyde oxime noscapine cause no infringements of Lipinski rule of five and chase all the obligatory properties desirable for a potent drug. These results suggested that owing to strong hydrophobicity, compounds will display long circulation in systemic circulation which will ultimately enhance the interactions with target protein tubulin. Pharmacokinetics data suggested high gastrointestinal absorption with no or minimal permeation to the blood brain barrier for both drugs (Table 2). Both compounds were found not found to inhibit the cytochrome (CYP)A2 and C19, this property will help in metabolism of drug with maintaining the homeostasis of the body fluids. Skin permeation score come out to be −7.71 cm/s and −7.36 cm/s for 9-methyl hydroxy noscapine and 9-oxime noscapine respectively. Notably compounds were also not found the substrate of Pgp substrate so these will stay longer in body. In view of medicinal properties, both compounds were also not found associated with side effects or pain. Hence, *in silico* docking analysis indicated that 9-hydroxy methyl and 9-carbaldehyde oxime analogues of Nos were more potent than the parent compound, Nos.

**Table 1:** Physicochemical and tubulin binding attributes of noscapine, 9-hydroxy methyl and 9- carbaldehyde oxime analogue of noscapine.

S.No.	Physicochemical and tubulin Properties	Noscapine	9-Hydroxy methyl analogue of noscapine	9-Carbaldehyde oxime analogues of noscapine
1	Binding Energy (KJ/mol)	−10.20	−10.73	−10.83
2	Inhibitory Constant (KI) (µM) [Temp = 298.15 K]	17.8	43.7	31.9
3	Molecular formula	C22H23NO7	C23H25NO8	C23H24N2O8
4	Molecular weight	413.15	443.16	456.15
5	Number of HBA	8	9	10
6	Number of HBD	0	1	1
7	MolLogP	2.36	1.54	2.16
8	MolLogS	−2.99 (in Log(mols/L)) 423.39 (mg/L)	−2.74 (in Log(mols/L)) 811.87 (mg/L)	−3.30 (in Log(mols/L)) 227.16 (mg/L)
9	MolPSA	64.77 A^2^	81.57 A^2^	93.47 A^2^
10	MolVol	412.13 A^3^	443.05 A^3^	451.60 A^3^
11	Nos. of stereocentres	2	2	2

**Table 2:** pharmacokinetic properties of noscapine derivatives 9-hydroxy methyl and 9- carbaldehyde oxime.

	9- methyl hydroxy noscapine	9-oxime noscapine
***Pharmacokinetics***		
GI absorption	High	High
BBB permeant	no	no
P-gp substrate	no	no
CYP1A2 inhibitor	no	no
CYP2C19 inhibitor	no	no
CYP2C9 inhibitor	yes	yes
CYP2D6 inhibitor	yes	yes
CYP3A4 inhibitor	yes	yes

### 9-hydroxy methyl and 9-carbaldehyde oxime analogues of noscapine induced higher extent of cytoxicity in U87 glioblastoma cells and U251 resistant glioblastoma cells

**Figure 5:**
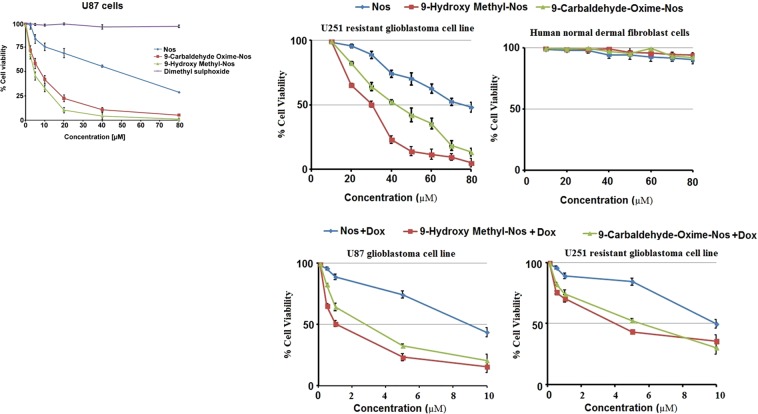
*In vitro* cytototoxicity analysis of noscapine (Nos), 9-Hydroxy methyl analogue of noscapine, and 9-carbaladehyde oxime analogue of noscapine against sensitive U87 glioblastoma cells in addition to resistant U251 glioblastoma cell line and human normal dermal fibroblast cell line either alone or in combination with doxorubicin.

Glioma is the most common brain tumor and has an undesirable prognosis due to the blood-brain barrier (BBB) and drug resistance. The over-expression of P-gp (P-glycoprotein) and MDR1 (Multidrug resistance gene 1) is associated with poor prognosis and drug-resistance in glioma cells. U251 glioblastoma cell line over-express P-gp and on the other hand, U87 displayed the expression of MDR-1^31^. Therefore both U87 and U251 cell lines were taken to investigate the anticancer activity. The therapeutic efficacy of 9-hydroxy methyl and 9-carbaldehyde oxime analogues of Nos was measured in terms of IC50 value and degree of apoptosis induced in U87 cells, respectively by standard cell proliferation assay^24^ and apoptosis assay^32^. The IC50 of Nos was estimated to be 46.8 µM significantly (One way ANOVA test, P < 0.05) higher than 8.2 µM of 9-carbaldehyde oxime and 4.6 µM of 9-hydroxy methyl analogue of Nos. Moreover, there was significant (Two-way ANOVA test, P < 0.05) difference between the IC50 of 9- carbaldehyde oxime and 9-hydroxy methyl analogue of Nos. Correspondingly, the IC50 of 9-hydroxy methyl analogue of Nos was estimated to be 32.6 µM significantly (One way ANOVA test, P < 0.05) lesser than 42.9 µM of 9-carbaldehyde oxime as well as 75.4 µM of Nos against U251 resistant glioblastoma cells. Although, none of the compounds displayed toxicity against human normal dermal fibroblast cells. On the other hand, a separate combination of Dox with Nos (50:50), 9-hydroxy methyl analogue of Nos (50:50) and 9-carbaldehyde oxime analogue of Nos (50:50) against U87 glioblastoma cells and U251 glioblastoma cells exhibited strong anticancer synergy **(**Fig. 5**)**. Regardless of its outstanding anticancer bustle, Dox has a comparatively squat therapeutic index and its clinical usefulness is limited owing to acute and chronic toxicities like dose-cumulative cardiotoxicity, myelosuppression, and immunosupression^33^. Hence, alliance with other chemotherapeutic agents may lesser the dose-dosage regimen.

The IC50 of 9-hydroxy methyl analogue of Nos + Dox was estimated to be 0.88 µM significantly (One-way ANOVA test, P < 0.05) lesser than 2.6 µM of 9-carbaldehyde oxime + Dox as well as 8.7 µM of Nos + Dox in U87 glioblastoma cells. Correspondingly, the IC50 of 9-hydroxy methyl analogue of Nos + Dox was estimated to be 3.9 µM significantly (One-way ANOVA test, P < 0.05) lesser than 5.3 µM of 9-carbaldehyde oxime + Dox as well as 9.9 µM of Nos + Dox against U251 resistant glioblastoma cells.

### 9-hydroxy methyl and 9-carbaldehyde oxime analogues of noscapine induced higher extent of apoptosis in U87 glioblastoma cells

**Figure 6:**
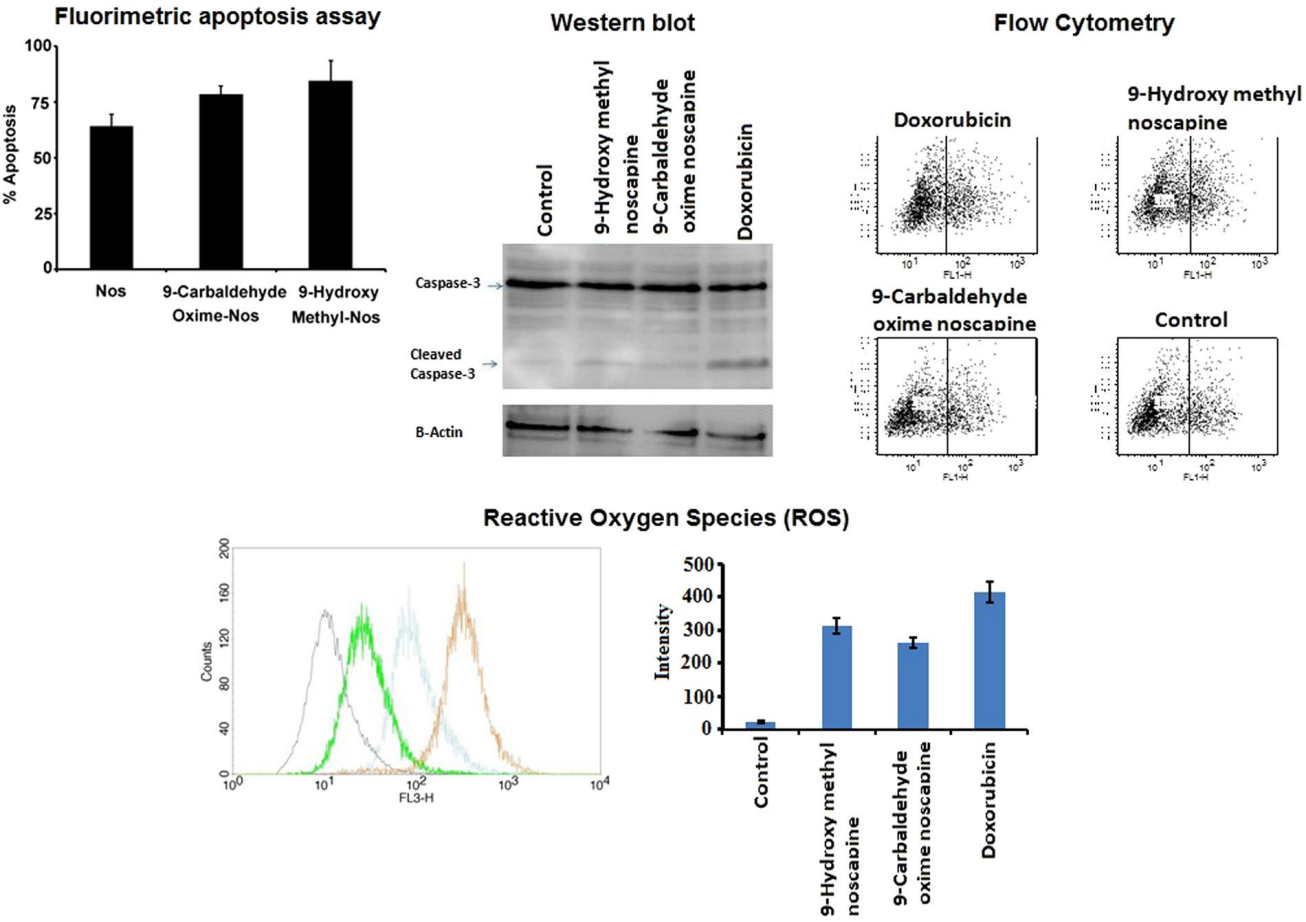
Measurement of degree of apoptosis using fluorimetric caspase-3 apoptosis assay kit, western blotting, and flow cytometry in addition to Reactive oxygen species (ROS) level in U87 glioblastoma cells.

Consistent to *in vitro* cytotoxicity data, 9-hydroxy methyl analogue of Nos provoked significantly (One-way ANOVA test, P < 0.05) elevated intensity of apoptosis of 84.6% in U87 cells in comparison to 78.5% and 64.3% demonstrated by 9-carbaldehyde oxime and Nos, respectively. In this way, *in vitro* cytotoxicity data and apoptosis assay supported the *in silico* docking predictions and informed that 9-hydroxy methyl analogue of Nos was more potent than 9-carbaldehyde oxime derivative (Fig. 6**)**. The extent of apoptosis was also confirmed by Western blotting, flow cytometry and ROS level. 9-hydroxy methyl analogue of Nos displayed 2.18-fold caspase-3 cleavage as compared to 1.64- fold demonstrated by 9-carbaldehyde oxime analogue of Nos (Fig. 6 and Suppl. Fig. 1). Similarly, 9-hydroxy methyl analogue of Nos induced 81.4% of apoptosis in U87 cells significantly (Unpaired t test, P < 0.05) higher than 72.3% of apoptosis induced by 9-carbaldehyde oxime analogue of Nos in flow cytometry analysis. On the other hand, doxorubicin was found to be highly toxic and induced 93.7% of apoptosis in U87 cells. The ROS level was measured to explore the mitochondria mediated apoptosis pathway. 9-hydroxy methyl analogue of Nos induced generated higher extent of ROS level in U87 cells as compared to 9-carbaldehyde oxime analogue of Nos (Fig. 6). Hence, 9-hydroxy methyl and 9-carbaldehyde oxime analogues were more effective than the parent compound, Nos. Previous reports indicated that improvement in the solubility of noscapinoids augmented the therapeutic index and consequently the anticancer potential^34–36^. Therefore, higher therapeutic efficacy of 9-hydroxy methyl analogue of Nos may be credited to higher solubility and inhibitory constant (K). The higher solubility of 9-hydroxy methyl analogue of Nos would have allowed the better penetration of therapeutic moiety across the cellular membrane of cancer cells that subsequently promoted the higher degree of cytotoxicity and apoptosis.

## Materials and Methods

### Materials

Noscapine [(3*S*)-6,7-Dimethoxy-3-((5*R*)-5,6,7,8-tetrahydro-4-methoxy-6-methyl-1,3-dioxolo (4,5-*g*) isoquinolin -5-yl)- 1 (3*H*) - isobenzofuranone] was purchased from Sigma-Aldrich, USA.

Formaldehyde, sodium carbonate, dichloromethane, and anhydrous sodium sulphate were procured from Merck, Mumbai, India. All other reagents and solvents used were of highest analytical grade.

### Cell culture

Human glioblastoma cell line, U87, U251 resistant glioblastoma cells and human normal dermal fibroblast cells were maintained as monolayers at 37 °C in 25 cm^2^ tissue culture flask (Tarsons, India) using Dulbecco’s Modified Eagle’s Medium (DMEM, Himedia, Mumbai, India) supplemented with 5% fetal calf serum (Biologicals, Israel). All assays were performed with asynchronous cell populations in exponential growth phase (24 h after plating)^37^.

### Instrumentation

FT-IR spectra were captured using Perkin Elmer spectrophotometer by forming a pellet of potassium bromide for solid samples. Melting point was determined using a Thomas-Hoover melting point apparatus. ^1^H NMR and ^13^C NMR spectra were captured using BRUKER AVANCE 300 NMR spectrometer in CDCl3. Tetramethylsillane was used as internal standard for ^1^H NMR and ^13^C NMR, respectively unless otherwise specified (Abbreviations for signal coupling are as follows: s, singlet; d, doublet; t, triplet; q, quartet; m, multiplet). Mass spectrum was recorded on Hybrid-Quardrupole-TOF LC/MS/MS mass spectrometer (Q. Star XL). All chemical reactions were conducted with oven dried (125 °C) reaction vessels. The reactions were monitored by thin-layer chromatography (TLC) using silica gel 60 F254 (Merck, Germany) mounted pre-coated aluminum sheets.

### Methodology

#### Synthesis of 3-(9-Hydroxymethyl-4-methoxy-6-methyl-5,6,7,8-tetrahydro-[1,3] dioxolo [4,5-g] isoquinoline-5-yl)-6,7-dimethoxy-3H-isobenzofuran-1-one

The synthesis of 9-hydroxy methyl analogue of Nos was carried out by Blanc reaction^26^. In brief, Nos (**Compound 1**) (0.413 g, 1 mM) was dissolved in concentrated HCl (5 ml) and then 40% formalin solution (5 ml) was added. The reaction mixture was stirred at room temperature for 15 min followed by cooling at 0 °C and a brisk stream of dry HCl gas was passed for 1 h. Cold solution was poured in crushed ice and made alkaline (pH~9) by using saturated sodium carbonate solution. The white precipitates were filtered, taken up into dichloromethane and washed with distilled water and brine, and dried over anhydrous sodium sulphate. The organic layer was evaporated to get the pure compound as white precipitates. The yield of 9-hydroxy methyl analogue of Nos was estimated to be 90%. The synthesis of 9-hydroxy methyl analogue of Nos (**Compound 2**) was confirmed by using FT-IR, ^1^H NMR, ^13^C NMR and mass spectroscopy.

FT-IR: 3520, 2950, 2852, 1751, 1635, 1450, 1267, 1226, 1078, 1035 cm^−1^; ^1^H NMR (300 MHz, CDCl3): δ = 7.25 (d, 1 H, J = 8 Hz), 6.27(d, 1 H, J = 7.5 Hz), 6.0 (s, 2 H), 5.48 (d, 1 H), 4.85(s, 1 H), 4.39 (s, 2 H), 4.23 (s, 1 H), 3.90 (s, 3 H), 3.87 (s, 3 H), 3.80 (s,3 H), 2.41(s,3 H), 2.54–1.93 ppm (m,4 H); ^13^C NMR (100 MHz, CDCl3): δ = 167.1, 152.1, 148.1, 146.6, 140.1, 133.9, 131.4, 119.0, 117.9, 116.5, 102.4, 100.9, 80.9, 61.5, 60.5, 59.3, 56.5, 53.8, 49.0, 45.6, 26.8 ppm; HRMS (ESI): m/z calculated for C23H25NO8 (M + 1): 444.165, measured: 444.1 (M + 1).

#### Synthesis of 5-(4,5-Dimethoxy-3-oxo-1,3-dihydro-isobenzofuran-1-yl)-4-methoxy-6-methyl-5,6,7,8-tetrahydro-[1,3]dioxolo[4,5-g]isoquinoline-9-carbaldehyde

The synthesis of intermediate carbaldehyde analogue of Nos (**Compound 3**) was carried out by oxidation reaction^27^. In brief, compound **2** (1 mM, 0.443 g) was dissolved in chloroform and then MnO2 was added in small portions with constant stirring. After the addition of MnO2, the suspension was refluxed for 4 h and then cooled and filtered. The filtrate obtained was evaporated to get the yellow product, which was purified by column chromatography. The estimated yield of **compound 3** was 70%. The melting point of **compound 3** was measured in the range of 169.2–170 °C. The synthesis of **compound 3** was confirmed using FT-IR, ^1^H NMR, ^13^C NMR, mass spectroscopy as well as elemental analysis. FT-IR: 2950, 2852, 1751, 1677, 1618, 1496, 1269, 1078, 1035 cm^−1^; ^1^H NMR (300 MHz, CDCl3): δ = 10.16 (s, 1H), 7.30 (d, 1H, J = 6.5), 6.57 (d, 1H, J = 6.96), 6.0 (s, 2H), 5.51 (d, 1H), 4.39 (s, 2H), 4.20 (s, 1H), 3.98 (s, 3H), 3.87 (s, 3H), 3.82 (s, 3H), 2.41 (s, 3H), 2.54–1.93 ppm (m, 4H); ^13^C NMR (CDCl3, 100 MHz): δ = 186.86, 67.05, 152.86, 146.68, 144.33, 141.14, 133.39, 129.11, 119.39, 117.87, 10.93,102.39, 100.85, 61.46, 60.54, 59.38, 57.83, 46.97, 45.30, 44.41, 22.69; (+TOF) MS: 441.93(M + 1); Analytical calculation for C23H23NO8: C 62.57, H 5.25, N 3.17; Measured: C 62.53, H 5.22, N 3.19.

#### Synthesis of 5-(4,5-Dimethoxy-3-oxo-1,3-dihydro-isobenzofuran-1-yl)-4-methoxy-6-methyl 5,6,7,8-tetrahydro-[1,3]dioxolo[4,5-g]isoquinoline-9-carbaldehyde oxime

The synthesis of 9-carbaldehyde oxime analogue of Nos was carried out with the method of oxime formation^28^. In brief, **compound 3** (1 mM, 441 mg) was dissolved in ethanol and then distilled water (2 ml), ice (500 g), and hydroxylamine HCl (500 mg) were added and the temperature was maintained at 0 °C for 15 min with constant stirring. After 15 min, 2 ml of 2 M NaOH was incorporated and the solution was stirred at room temperature overnight. The solution was made alkaline (pH~9) with 2 M NaOH and extracted with diethyl ether to remove unreacted portions. Aqueous phase was acidified with HCl to maintain the pH 6 and then extracted with chloroform. The chloroform was evaporated to get the white crystals of **compound 4**. The yield of **compound 4** was estimated to be 75%. The melting point of **compound 4 (**9-carbaldehyde oxime**)** was measured in the range of 120.3–120.9 °C. The synthesis was confirmed using FT-IR, ^1^H NMR, ^13^C NMR, mass spectroscopy and elemental analysis. FT-IR: 2950, 2852, 1751, 1635, 1450, 1267, 1226, 1078, 1035 cm^−1^; ^1^H NMR (300 MHz, CDCl3): δ = 11.30(s, 1H), 7.30(d, 1H, J = 7.5), 6.27(d, 1H, J = 7.38), 6.06(s, 2H), 5.51(d, 1H,), 4.25(s, 1H,), 3.93(s, 3H), 3.87(s, 3H), 3.80(s, 3H), 2.41(s, 3H), 2.54–1.93 ppm (m, 4H); ^13^C NMR (CDCl3, 100 MHz):δ = 167.10, 152.05, 147.13, 142.82, 140.87, 140.40, 133.55, 129.73, 119.24, 117.89, 117.23, 107.19, 101.32, 80.74, 61.48, 60.69, 59.31, 56.59, 48.14, 45.03, 28.97, 24.41 ppm; (+TOF) MS: 456.92 (M + 1); Analytical calculations for C23H24N2O8: C: 60.52, H: 5.29, N: 6.13, Measured: C: 60.22, H: 5.35, N: 5.92.

### *In silico* docking

The 9-hydroxy methyl and 9-carbaldehyde oxime analogues of Nos (**Compound 2 and 4**) were further evaluated using *in silico* docking technique to determine the physicochemical and anticancer attributes. Both compounds were first docked with Autodock 4.0 and then studied for Lipnski rule of five using Molinspiration and pkCSM web interfaces^38^. Autodock 4.0 server is a protein-ligand docking analysis module. Autodock ligand preparation module utility generates new structure from each input structure with various ionization states, tautomers, stereochemistries, and ring conformations^39^. All possible conformations were derived through the program and unique low-energy ring conformations were estimated. Tubulin receptor protein was retrieved from Protein Data Bank in PDB format and evaluated by its Ramachandran plot to analyze its stereochemical properties using Rampage server^30^. Lead compounds 2 and 4 were drawn using the Chemdraw and saved in mol2 format.

### Therapeutic efficacy testing of synthesized compounds

#### Standard cell proliferation assay

*In vitro* anticancer activity of compound 2 and 4 was determined by standard cell proliferation assay^24^ using a 96-well microtiter plate. In brief, In brief, 5 × 10^3^ U87 cells were plated in 200 µl of DMEM medium. After 24 h, the DMEM medium was removed and replaced with a gradient concentration (10–80 µM) of Nos (**Compound 1**), 9-hydroxy methyl-Nos (**Compound 2**) and 9-carbaldehyde oxime-Nos (**Compound 4**) dissolved in DMSO (Dimethyl sulphoxide) and incubated for 24 h. At the end of treatment, U87 cells were treated with MTT (0.5 mg/ml) in dark for 4 h at 37 °C. In last, medium was removed and formazan crystals were dissolved by using 100 µl of DMSO. The absorbance was read at 570 nm in an ELISA plate reader (Tecan, Switzerland). The experiment was performed in triplicate (n = 3). Correspondingly, *in vitro* anticancer activity of Nos, 9-hydroxy methyl-Nos and 9-carbaldehyde oxime-Nos was also measured against U251 resistant glioblastoma cells and human normal dermal fibroblast cells, respectively in addition to combination with 50% doxorubicin (Dox).

#### Apoptosis assay

The extent of apoptosis induced by compound 2 and 4 in U87 cells was measured by fluorometric caspase-3 apoptosis assay kit^32^ as per the manufacturer’s instructions (Merck Millipore). In brief, 3 × 10^5^ U87 cells were incubated with 20 µM of Nos or 9-hydroxy methyl-Nos or 9-carbaldehyde oxime-Nos for 24 h separately in a sterile polystyrene petridish (Tarson, India) of 35 mm diameter. The treated cells were collected in a pellet form at the end of treatment and resuspended in 50 µl of cell lysis buffer. The cell lysis buffer was then incubated for 10 min on ice. Subsequently, centrifugation of cells was carried out at 2800x g for 10 min, and the supernatant was transferred in to a 96-wells microtitre plate to which 50 µl of reaction buffer (50 mM/L PIPES, pH~7.4, 10 mM/L EDTA, 0.5% CHAPS) containing 10 mM/l dithiothreitol and 5 ml of the respective substrate was added as per the protocol. The plate was left for 1 h at room temperature and fluorescence was measured in a fluorometer (exe~400 nm, emi~505 nm). The protein content was estimated using BCA protein assay kit^40^. The experiment was performed in triplicate (n = 3).

#### Western blot analysis

U87 glioblastoma cells were treated with noscapine (20-µM), 9-hydroxy methyl analogue of Nos (Compound 2, 20-µM), 9-carbaldehyde oxime analogue of Nos (Compound 4, 20-µM) and Dox (10-µM) for 48 h. Cells were lysed in RIPA buffer, separated by PAGE on 15% w/v SDS-PAGE gel. β-actin (42 kDa) was used as a reference owing to its expression in almost all eukaryotic cells as well as unaltered post treatment expression. Later, the gel was electrophoretically transferred to a PVDF membrane (Millipore, Billerica, Massachusetts, USA). The blotted membrane was incubated at room temperature for 2 h with 5% nonfat milk. The membrane was initially stained with primary antibodies against β-actin and caspase-3 overnight at 4 °C, and then incubated with fluorescent labelled goat anti-mouse secondary antibody or goat anti-rabbit secondary antibody^41^. The bands were then detected using an enhanced chemiluminescence (ECL) detection system (Pierce, Rockford, IL, USA). The quantification of the bands was carried out using the gel analysis submenu of Image J software.

#### Flow cytometry analysis

The extent of apoptosis was also analyzed using standard fluorescence-activated cell sorting (FACS) assay^32^. In brief, 40 × 10^4^ U87 cells were treated with noscapine (20-µM), 9-hydroxy methyl analogue of Nos (Compound 2, 20-µM), 9-carbaldehyde oxime analogue of Nos (Compound 4, 20-µM) and Dox (10-µM) for 48 h. The cells were then gathered with trypsin-EDTA, centrifuged (Remi, Mumbai, India) and washed twice with ice-cold PBS. Following this, binding buffer (1×) was added to each tube to make the final concentration of 4 × 10^4^ cells/ml.

Subsequently, 100 µl of U87 cell suspension from each tube was taken in to 5 ml capacity FACS tube. Finally, 5 µl PE Annexin V + 5 µl 7-AAD was added to all FACS tubes. Cells were vortexed and incubated for 15 min at room temperature. After mixing, 400 µl of 1x binding buffer was added to each tube before analyses on a FACSCalibur flow cytometer (Beckman Coulter, Inc., Fullerton, CA). The extent of apoptosis (in percent) was assessed by the formula:

$$\frac{ \% \,{\rm{Apoptosis}}=({\rm{Cell}}\,{\rm{viability}}\,{\rm{in}}\,{\rm{control}}\,{\rm{group}} \mbox{-} {\rm{cell}}\,{\rm{viability}}\,{\rm{in}}\,{\rm{drug}}\,{\rm{treated}}\,{\rm{group}})\times 100}{{\rm{Cell}}\,{\rm{viability}}\,{\rm{in}}\,{\rm{control}}\,{\rm{group}}}$$

At least 10,000 cells were characterized by flow cytometry for apoptosis analysis. All tests were performed in doublets.

#### Reactive oxygen species (ROS) level

The levels of ROS were measured according to previously published method using DCF-DA (Sigma-Aldrich, USA^42^. In brief, U87 cells were treated with noscapine (20 µM), 9-hydroxy methyl analogue of Nos (Compound 2, 20-µM), 9-carbaldehyde oxime analogue of Nos (Compound 4, 20-µM) and Dox (10-µM) for 12 h. Then, the cells were collected, washed with saline and resuspended in tubes with saline containing 5 μM DCF-DA for 30 min. Finally, the cells were washed with saline and the cell fluorescence was determined by plate spectrofluorometer (Horiba, Japan).

### Statistical analysis

Results were expressed as mean ± standard deviation (mean ± S.D.). Statistical difference was analyzed with one-and two-way analysis of variance tests as well as student t test. P < 0.05 was considered to be statistically significant.

## Conclusion

In conclusion, the analogues, 9-hydroxy methyl and 9-carbaldehyde oxime of Nos can be easily scaled up with high yield using simple and reproducible synthetic protocols. Moreover, 9- hydroxy methyl and 9-carbaldehyde oxime analogues of Nos have demonstrated potent anticancer activity in terms of high therapeutic index and tubulin binding constant as compared to parent compound, Nos through *in silico* studies. In addition, 9-hydroxy methyl analogue of Nos owing to its high solubility at physiological pH displayed higher therapeutic efficacy as compared to 9-carbaldehyde oxime analogue of Nos through the pharmacological and pharmacokinetics studies. Therefore, both 9-hydroxy methyl and 9-carbaldehyde oxime analogues of Nos warrant further in depth *in vitro* and *in vivo* study under a set of stringent parameters for translating in to the clinically viable products.

## Supplementary information

Supplementary Information 
