# Supplementary material for: Biological Evaluation of Noscapine analogues as Potent and Microtubule-Targeted Anticancer Agents
Source: Sci Rep. 2019 Dec 20;9:19542. doi: 10.1038/s41598-019-55839-8 (PMC6925231; doi:10.1038/s41598-019-55839-8)
Supplement: Supplementary file 1 — Supplementary Information [file 41598_2019_55839_MOESM1_ESM.docx]

**Biological Evaluation of Noscapine analogues as Potent and Microtubule-Targeted Anticancer Agents**

Vartika Tomar^1,2^, Neeraj Kumar^1^, Ravi Tomar^1^, Damini Sood^1^, Neerupma Dhiman^3^, Sujata K. Dass^4^, Satya Prakash^2^, Jitender Madan^5^ and Ramesh Chandra^1,6*^

*^1^Department of Chemistry, University of Delhi, Delhi, India*

*^2^BioMedical Engineering Department, Faculty of Medicine, McGill University, Montreal, Canada*

*^3^Amity Institute of Pharmacy, Noida (U.P), India*

*^4^BL Kapur Hospital, New Delhi-110005*

*^5^Chandigarh College of Pharmacy, Mohali (Panjab) India*

1. *Dr .B. R. Ambedkar Center for Biomedical Research, University of Delhi, Delhi, India*

**________________________________________________________________________**

*****Corresponding author: Prof. Ramesh Chandra, Drug Discovery & Development Laboratory, Department of Chemistry, University of Delhi and Dr.B.R.Ambedkar Center for Biomedical Research, UniversityofDelhi, Delhi- 110007, India

Tel: + 91-11-27666245, 27667151; Fax: +91-11-27666294

Email: acbrdu@hotmail.com, rameshchandragroup@gmail.com


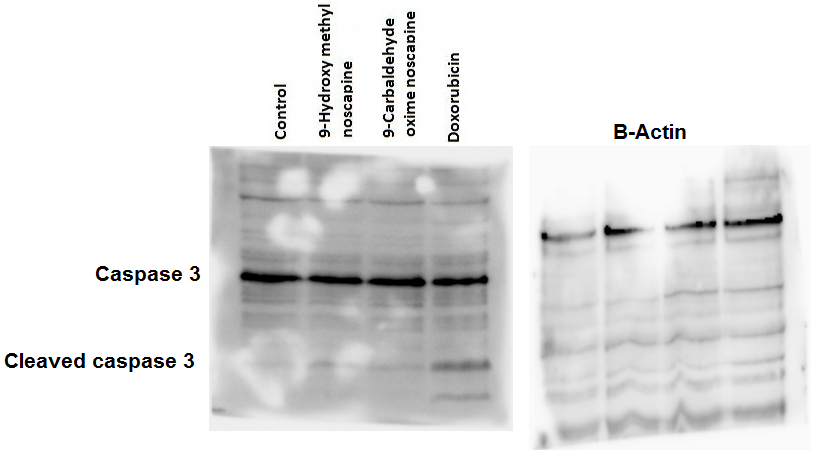


**Suppl. Figure 1:** Full length Western blot of Caspase 3 assay with B-Actin as reference
